# A complete extracorporeal circulation free approach to patients with univentricular hearts provides superior early outcomes

**DOI:** 10.1186/1749-8090-8-S1-O313

**Published:** 2013-09-11

**Authors:** Sachin Talwar, S Muthukkumaran, SK Choudhary, N Makhija, V Sreeniwas, A Saxena, R Juneja, SS Kothari, B Airan

**Affiliations:** 1 Department of Cardiothoracic and Vascular Surgery, All India Institute of Medical Sciences, New Delhi -110029, India; 2 Cardiothoracic Sciences Centre, All India Institute of Medical Sciences, New Delhi-110029, India, New Delhi, India; 3 Department of Biostatistics All India Institute of Medical Sciences, New Delhi-110029, India, New Delhi, India; 4 All India Institute of Medical Sciences, New Delhi-110029, India, New Delhi, India

## Background

Although there are small reported series of patients undergoing the Extracardiac Total cavopulmonary connection without cardiopulmonary bypass, there is paucity of data comparing the results of the two.

## Methods

Between February 2012 and February 2013, 27 patients undergoing Total cavopulmonary connection without cardiopulmonary bypass (off-pump group) were compared with propensity score matched 27 patients undergoing Total cavopulmonary connection on cardiopulmonary bypass (on-pump group). Outcome parameters studied were inotropic score, time to extubation, intensive care unit stay, mediastinal drainage in the intensive care unit, daily pleural drainage, time to removal of chest tubes, hospital stay and saturation at discharge.

## Results

There was one early death in each group. No patient required conversion from off pump to cardiopulmonary bypass. The inotropic score (6.1±5.91versus 10.1±6.80, p=0.03), time to extubation (8.7±6.95versus 10.31±8.69hours, p=.03), mediastinal drainage in intensive care unit (611.9±341.4versus 922.2±145.6 ml,p=0.03) and intensive care unit stay (1.6±0.58 versus 2.9±1.37days,p<0.001) were significantly less in off pump group as compared to on-pump group and saturation at discharge (99.7±0.60 versus 98.6±2.13, p=0.026) was higher in the off-pump group. However the daily pleural drainage (125±61.7.2versus 150±103.4ml, p=0.7), time to removal of chest tubes (12.69±7.1 versus 15.44±19.26 days, p=0.45) and the total hospital stay (14.23±7.4 versus 18.89±19.9 days, p=0.22) were no different. There were substantial savings in costs in patients undergoing off-pump procedure.

## Conclusion

Extracardiac Total cavopulmonary connection without cardiopulmonary bypass is easy to perform, is cost effective, and is associated with superior early post-operative outcomes. With appropriate modifications, this operation can be performed in almost all morphological subsets of patients who do not need an associated intracardiac procedure.

